# Trends in Concurrency, Polygyny, and Multiple Sex Partnerships During a Decade of Declining HIV Prevalence in Eastern Zimbabwe

**DOI:** 10.1093/infdis/jiu415

**Published:** 2014-12-01

**Authors:** Jeffrey W. Eaton, Felicia R. Takavarasha, Christina M. Schumacher, Owen Mugurungi, Geoffrey P. Garnett, Constance Nyamukapa, Simon Gregson

**Affiliations:** 1 Department of Infectious Disease Epidemiology, Imperial College London, United Kingdom; 2 Biomedical Research and Training Institute; 3 AIDS and TB Unit, Zimbabwe Ministry of Health and Child Welfare, Harare, Zimbabwe; 4 Department of Pediatrics, Johns Hopkins University School of Medicine, Baltimore, Maryland; 5 Global Health Program, Bill and Melinda Gates Foundation, Seattle, Washington

**Keywords:** HIV, Zimbabwe, behavior change, multiple partnerships, polygyny, concurrency, divorce

## Abstract

***Background.*** Observed declines in the prevalence of human immunodeficiency virus (HIV) infection in Zimbabwe have been attributed to population-level reductions in sexual partnership numbers. However, it remains unknown whether certain types of sex partnerships were more important to this decline. Particular debate surrounds the epidemiologic importance of polygyny (the practice of having multiple wives).

***Methods.*** We analyze changes in reported multiple partnerships, nonmarital concurrency, and polygyny in eastern Zimbabwe during a period of declining HIV prevalence, from 1998 to 2011. Trends are reported for adult men (age, 17–54 years) and women (age, 15–49 years) from 5 survey rounds of the Manicaland HIV/STD Prevention Project, a general-population open cohort study.

***Results.*** At baseline, 34.2% of men reported multiple partnerships, 11.9% reported nonmarital concurrency, and 4.6% reported polygyny. Among women, 4.6% and 1.8% reported multiple partnerships and concurrency, respectively. All 3 partnership indicators declined by similar relative amounts (around 60%–70%) over the period. Polygyny accounted for around 25% of male concurrency. Compared with monogamously married men, polygynous men reported higher levels of subsequent divorce/separation (adjusted relative risk [RR], 2.92; 95% confidence interval [CI], 1.87–4.55) and casual sex partnerships (adjusted RR, 1.63; 95% CI, 1.41–1.88).

***Conclusions.*** No indicator clearly dominated declines in partnerships. Polygyny was surprisingly unstable and, in this population, should not be considered a safe form of concurrency.

Zimbabwe, where the prevalence of human immunodeficiency virus (HIV) infection among adults is estimated to have declined from a peak of 27.6% in 1997 to 14.7% in 2012 [1], has been one of the most successful countries in sub-Saharan Africa in reducing the spread of HIV [2]. This decline in prevalence followed sustained declines in the incidence of HIV infection since the early 1990s [1, 2]. The contributions of reductions in numbers of sex partners to reductions in HIV incidence in Zimbabwe [2–4] and elsewhere [5, 6] are well documented, but there remains debate about the relative importance of reductions of different types of partnerships to controlling HIV epidemics, particularly multiple exclusive partnerships versus nonexclusive concurrent (ie, overlapping) partnerships [7, 8]. This has important implications for the optimal design and messaging of HIV prevention [9].

Polygyny, the practice of men having multiple wives, is culturally acceptable and common across sub-Saharan Africa [10]. The role of polygyny in the spread of HIV has been greatly debated. Polygynous marriages are an institutionalized form of concurrent sexual partnerships [10], which theoretically can increase the spread of HIV by creating more connected sexual networks enabling more-rapid onward transmission of the virus [11–13]. Alternatively, it has been suggested that polygynous marriages are likely to be more stable than informal concurrency [10] and that strictly sex-asymmetric concurrency, in which only men have multiple wives, creates isolated sexually connected groups (eg, triads) that are no more risky than faithful monogamous partnerships [14, 15]. In light of this, some have argued that targeting polygyny as a form of concurrency could be a counterproductive imposition of Western norms [16] and that, for public health messaging and policy, risky nonmarital concurrency should be measured and considered separately from polygyny.

Whether polygyny should be distinguished from nonmarital concurrency in HIV prevention policy should depend, in part, on empirical evaluation of levels of sexual risk behavior exhibited by polygynously married men, compared with that among monogamously married men and those in nonmarital concurrent partnerships. We use data collected in rural eastern Zimbabwe from 1998 through 2011 to evaluate the relative population-level changes in different types of sex partnerships—multiple sexual partnerships (as a whole), concurrency, and polygyny—during this period of declining HIV prevalence. If polygyny is a more stable and institutionalized form of concurrency, then it might be that there are larger declines in nonmarital concurrency while polygyny is more durable, accounting for a larger share of overall concurrency over time. Second, we evaluate the notion that polygyny represents a stable and safe form of concurrency by longitudinally comparing how polygynous, monogamous, or unmarried male marital status affects the risk of divorce or separation and having casual sex partnerships, each of which increase exposure to HIV transmission [17, 18].

## METHODS

### Study Population and Data Collection

The Manicaland HIV/STD Prevention Project is a population-based open cohort study in eastern Zimbabwe [3]. The study population consists of 12 geographically distinct communities in Manicaland Province (4 subsistence farming areas, 2 roadside trading settlements, 4 large-scale agricultural estates, and 2 rural commercial centers) with a total current population of around 57 000. The cohort was established from 1998 to 2000 and has completed 5 survey rounds, each occurring over 2–3 years. At each round, all households and residents were enumerated, and eligible adults (aged 17–54 years for men and 15–49 years for women) are invited to join the study. In the first 2 rounds, only 1 adult per marital relationship was randomly selected, to avoid nonindependence in the sample. In the third round, all adults were eligible, and in the fourth and fifth rounds, all adults were eligible but from only a randomly selected two-thirds of households, owing to funding constraints.

Study participants completed a face-to-face interview and donated blood specimens for anonymized dried blood spot testing. Information about demographic characteristics, marriage, and divorce was provided directly to the interviewer. Information about numbers of sex partnerships, concurrency, and casual sex partnerships was reported via informal confidential voting interview (ICVI) to ensure anonymity of responses. In the first round, respondents were randomized such that three-quarters gave sexual behavior information through ICVI and one-quarter answered directly [19]. In subsequent rounds, existing cohort members used the same interview method as the previous round, but all new members answered via ICVI. Thus, the percentage answering via ICVI increased to 82%, 89%, 91%, and 92% in rounds 2–5.

All respondents provided written informed consent at each survey round prior to completing the survey and providing a blood sample. Prior ethical approval for the Manicaland HIV/STD Prevention Project was granted by the Medical Research Council of Zimbabwe (Harare), the Applied and Qualitative Research Ethics Committee (Oxford University), and the St. Mary's Local Research Ethics Committee (Imperial College London).

### Sex Partnership Indicators

We investigate trends in 3 sex partnership indicators: multiple sex partners in the past year and the point prevalence of concurrency and polygyny [20]. Having multiple partnerships was determined on the basis of response to the question “How many different sex partners have you had in the last 12 months?” Concurrency and polygyny were measured at the time of the survey. Respondents with concurrent partnerships were those who stated that they had ≥2 such partnerships, in response to the question “How many sexual relationships do you consider yourself to be involved in at the moment?” Marriage was defined as being married or in a cohabiting relationship for >12 months. Polygynous men were identified as those reporting >1 such marital or long-term cohabiting relationship at the time of the survey.

For men, we separate all concurrency into polygyny and nonmarital concurrency. It is possible for a polygynous man to also exhibit nonmarital concurrency if he reports more current sex partners than reported wives (eg, reporting ≥3 current sexual relationships when he has 2 wives). Divorce between survey rounds was defined as reporting experiencing divorce or separation from a marital or cohabiting partner during the period since the previous survey. Having casual sex partners was defined as reporting ≥1 nonregular sex partner since the previous survey. Survey questionnaires are available at http://www.manicalandhivproject.org/questionnaires.html.

### Statistical Analysis

Trends are reported over the 5 survey rounds separately for men aged 17–54 years and women aged 15–49 years and by 5-year age groups (17–19 years for the youngest male age group). Exact binomial confidence intervals (CIs) are reported. We use log-binomial regression to compare the percentage reduction in each indicator in each round, compared with the baseline survey, from 1998 to 2000, with adjustment for 5-year age group, socioeconomic stratum (subsistence farming, roadside trading, agricultural estate, and commercial center), and religious affiliation (Christian, traditional, spiritual, other, and none [21]).

We longitudinally evaluated the association between male marital status (unmarried, monogamously married, and polygynously married) and subsequent divorce and acquisition of casual sex partners. For men followed up in the subsequent survey round, we use log-binomial regression to estimate the relative risk (RR) of divorce in the subsequent 3 years or reporting >1 casual sex partner in the intersurvey period, by marital status, with adjustment for age group, survey round, socioeconomic stratum, and religious affiliation. Information about divorce between survey periods was not available for the most recent survey (round 5), so analysis of divorce is restricted to men whose marital status was reported in rounds 1–3 (followed up in rounds 2–4). Analysis of casual partnerships is for men interviewed in rounds 1–4. For HIV-negative men, we used Poisson regression to estimate the HIV incidence rate ratio associated with marital status, adjusted for age group, survey round, and sociodemographic characteristics.

## RESULTS

Demographic characteristics of cohort participants in each round are summarized in Supplementary Table 1. The resident population has aged, with the mean age of both men and women increasing over time. The fraction of men living on agricultural estates declined over time, giving way largely to roadside trading areas and subsistence farming. The sex ratio remained similar across rounds. Around or just over half reported Christian religious affiliation, and men were more likely than women to report no religious affiliation. The fraction of married adults increased slightly. The estimated HIV prevalence declined between each survey round, from 19.7% in 1998–2000 to 13.5% in 2009–2011 for men and from 25.8% to 17.3%, respectively, for women.

### Trends in Multiple Sex Partnerships, Concurrency, and Polygyny

**Figure 1.**
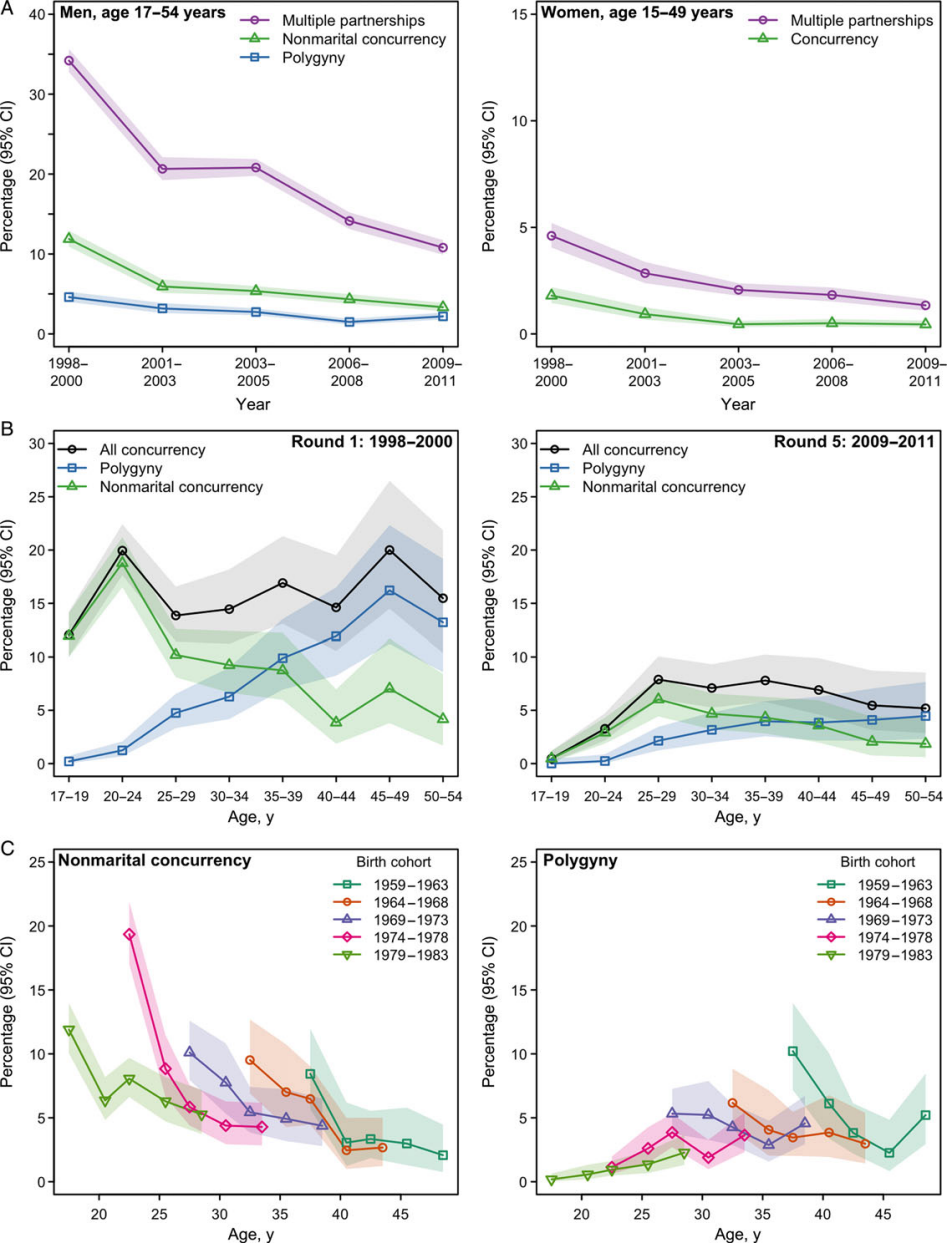
*A*, Trends is sex partnership indicators over 5 survey rounds (1998–2011) for men aged 17–54 years (left) and women aged 15–49 years (right). *B*, Patterns of male polygyny and nonmarital concurrency by five year age group at round 1 (1998–2000; left) and round 5 (2009–2011; right). *C*, Trend, by birth cohort, in male nonmarital concurrency (left) and polygyny (right), illustrating the levels of each type of sex partnership at the same age for successive cohorts. Abbreviation: CI, point-wise confidence interval.

At baseline, 34.2% of men (95% CI, 32.8%–35.7%) reported multiple partnerships in the past year, 11.9% (10.9%–12.9%) reported nonmarital concurrency, and 4.6% (4.0%–5.3%) reported polygyny. Among women, 4.6% (4.1%–5.2%) reported multiple partnerships, and 1.8% (1.5%–2.2%) reported concurrency. All of the indicators declined over time, with the relative levels remaining similar (Figure 1*A*). The most substantial decline occurred between the first 2 rounds, with continuing declines between each round, except for polygyny, which declined substantially between rounds 3 (2.7%; 95% CI, 2.3%–3.2%) and 4 (1.5%; 1.2%–1.9%) but recovered somewhat in round 5 (2.2%; 1.8%–2.7%). At baseline, polygyny accounted for approximately 24% of all concurrency (Supplementary Figure 1). This increased modestly over the survey rounds, to an estimated 35% in in the most recent round (Supplementary Figure 1), excepting the decline in polygyny in round 4.

The relative declines in each of the indicators were similar, with adjustment for age group, socioeconomic stratum, and religious affiliation (Supplementary Table 2). The proportion of men reporting multiple partnerships, nonmarital concurrency, and polygyny reduced by 37% (adjusted RR, 0.63; 95% CI, .58–.68), 50% (adjusted RR, 0.50; 95% CI, .42–.59), and 34% (adjusted RR, 0.66; 95% CI, .52–.83), respectively, between the first 2 survey rounds and by 66% (adjusted RR, 0.34; 95% CI, .31–.38), 70% (adjusted RR, 0.30; 95% CI, .25–.36), and 58% (adjusted RR, 0.42; 95% CI, .33–.53), respectively, by the most recent survey. For women, reporting of multiple partnerships and concurrency reduced by 31% (adjusted RR, 0.69; 95% CI, .56–.85) and 42% (adjusted RR, 0.58; 95% CI, .40–.83), respectively, between the first 2 surveys and by 68% (adjusted RR, 0.32; 95% CI, .26–.41) and 71% (adjusted RR, 0.29; 95% CI, .20–.44), respectively, in the most recent survey.

Both men and women in agricultural estates and especially commercial centers reported higher levels of multiple partnerships, concurrency, and polygyny than those in subsistence farming or roadside trading areas (Supplementary Table 2). Those reporting no religious affiliation reported higher levels of all indicators. Christian men reported lower levels of polygyny than men of other religions but not lower levels of nonmarital concurrency.

### Changes in Male Nonmarital Concurrency and Polygyny Over Age and Time

At baseline, levels of concurrency were high, between 12.0% and 20.0%, in all male age groups, but there were substantially different patterns for nonmarital concurrency and polygyny (Figure 1*B*). The prevalence of nonmarital concurrency was highest in the young age groups and accounted for almost all concurrency at these ages. It declined in older age groups. Polygyny exhibited the opposite pattern. It was very low among young men and highest for older men, accounting for 70% of concurrency in men aged >40 years. In the most recent survey, levels of concurrency were lower than at baseline in all male age groups, especially among those aged 17–24 years. However, the relative contributions of different forms of concurrency across age groups were similar to baseline values: nonmarital concurrency accounted for almost all concurrency among those aged <25 years, and polygyny accounted for almost all concurrency in the oldest age group (Figure 1*B*).

To determine whether reductions in concurrency represented cohort effects or behavior change across the population over the period, trends in nonmarital concurrency and polygyny by birth cohort were measured (Figure 1*C*). Nonmarital concurrency declined similarly over time within all birth cohorts (Figure 1*C*). Polygyny exhibited both effects (Figure 1*C*). In earlier birth cohorts (older at baseline), polygyny declined over time. More-recent birth cohorts did not report the same levels of polygyny experienced by earlier birth cohorts at the same ages.

### Polygyny and Risk of Divorce, Casual Sex Partnerships, and HIV Incidence

Polygynous men were more likely to become divorced and have had casual sex partnerships at the next survey round than monogamously married men (Table 1). Accordingly to crude data, 9.7% of polygynous men reported divorce at the next survey round, compared with 3.2% of monogamously married men and 2.6% of unmarried men. After adjustment for age group, survey round, socioeconomic stratum, and religion, polygynous men were 2.92 (95% CI, 1.87–4.55) times as likely to become divorced. After accounting for the number of spouses, the per-partnership RR of divorce was 1.26 (95% CI, .79–2.03) for polygynous men, compared with monogamous men.

**Table 1.** Risk of Divorce and Having Casual Sex Partners During the Intersurvey Period, by Marital Status at Baseline, Among Men Aged 17–54 Years

Characteristic		Divorce Between Surveys		Casual Sex Partners Between Surveys
	No. (%)	Adjusted RR (95% CI)^a^	No. (%)	Adjusted RR (95% CI)^a^
Age group, y				
17–19		0.41 (.20–.82)		0.91 (.82–1.01)
20–24		1.19 (.76–1.87)		1.17 (1.07–1.29)
25–29		Reference		Reference
30–34		1.09 (.68–1.75)		1.02 (.91–1.14)
35–39		0.94 (.56–1.58)		0.84 (.73–.96)
40–44		0.57 (.29–1.11)		0.75 (.64–.88)
45–49		0.76 (.40–1.42)		0.60 (.50–.73)
50–54		0.28 (.10–.79)		0.59 (.48–.72)
Survey interval				
Round 1 to 2		Reference		Reference
Round 2 to 3^b^		0.59 (.40–.86)		0.99 (.92–1.07)
Round 3 to 4		0.73 (.51–1.04)		0.84 (.77–.91)
Round 4 to 5		…		0.58 (.52–.64)
Socioeconomic stratum				
Subsistence farming		Reference		Reference
Roadside trading		1.32 (.78–2.22)		1.09 (.99–1.19)
Agricultural estate		1.16 (.77–1.74)		1.00 (.92–1.08)
Commercial center		1.58 (1.00–2.49)		1.12 (1.02–1.23)
Religious affiliation				
Christian		Reference		Reference
Traditional		1.13 (.70–1.83)		1.06 (.95–1.19)
Spiritual		1.16 (.75–1.79)		0.87 (.79–.96)
Other		1.08 (.48–2.45)		0.94 (.81–1.09)
None		1.55 (1.05–2.30)		1.12 (1.02–1.21)
Baseline marital status				
Unmarried	2427 (2.55)	0.87 (.58–1.29)	3080 (45.4)	1.52 (1.40–1.66)
Married	2776 (3.24)	Reference	3896 (24.7)	Reference
Polygynous	238 (9.66)	2.92 (1.87–4.55)	274 (41.2)	1.63 (1.41–1.88)

Abbreviations: CI, confidence interval; RR, relative risk.

^a^ Based on log-binomial model adjusted for all other covariates listed in the table.

^b^ The lower probability of divorce between rounds 2 and 3 is in part because there were only 2 years between rounds 2 and 3, compared with 3 years between each of the other survey rounds.

For casual partnerships, 41.2% of polygynous men and 45.4% of unmarried men reported casual partnerships, compared with 24.8% of monogamously married men. After adjustment for covariates, polygynous and unmarried men were 1.63 (95% CI, 1.41–1.88) times and 1.52 (95% CI, 1.40–1.66) times, respectively, more likely to have casual partners than monogamously married men. Twelve incident HIV infections were observed among polygynous men, for a crude incidence rate of 2.2 cases per 100 person-years, compared with 1.3 cases per 100 person-years (106 infections/8165 person-years) for monogamously married men and 1.1 (86 infections/7548 person-years) for unmarried men. After adjustment for age group, survey round, socioeconomic stratum, and religion, polygynous men had a risk for HIV infection that was 1.46 (95% CI, .80–2.68) times that for married men.

## DISCUSSION

There were substantial declines in all 3 sex partnership indicators during 1998–2011. The largest decline in each indicator occurred between the first 2 surveys, from 1998 to 2003, when incidence is also estimated to have declined most rapidly [1]. The relative changes in each of the indicators were similar for both men and women: each declined by 60%–70% since baseline, after adjustment for changes in the demographic composition. The prevalence of multiple partnerships was about 2.5 times that of concurrency for men and around 3 times that for women, leaving a substantial fraction of men who had multiple sex partnerships but were not involved in concurrent sexual relationships. Similarly, formal polygyny accounted for only one-quarter to one-third of all male concurrency.

At baseline, men of all ages reported concurrency, but the form of concurrency—nonmarital or polygyny—strongly depended on age. Nonmarital concurrency accounted for all concurrency in young men, giving way to polygyny in older men. Analysis by birth cohort illustrated that declines in polygyny were attributed to both more-recent birth cohorts not attaining the high levels of polygyny of previous cohorts and surprisingly steep declines in polygyny among older men, indicating a change in behavior over time across the whole population, rather than solely a cohort effect.

Our findings confirm previous research that men in polygynous unions have high rates of dissolution [22] and are more likely to have extramarital partners [23–25]. While this does not necessarily contradict the negative ecological association between levels of polygyny and HIV prevalence [10, 14, 25, 26], it challenges the sexual network hypothesis proposed to explain this: that polygynous partnerships are isolated network structures into which HIV infections are adversely selected and trapped from further onward transmission [14]. It remains possible that the negative correlations identified in ecological analyses are attributable to residual confounding by geographic, sociocultural, or epidemiological factors that were not included or able to be adequately adjusted for in linear models, rather than a true causal relationship between level of polygyny and HIV spread [27].

The reductions in polygyny in eastern Zimbabwe are consistent with documented declines in polygyny over the past few decades across sub-Saharan Africa [28]. While previous research has concluded that reductions in sex partners in Zimbabwe was a response to awareness of the devastating effects of HIV and AIDS [2], it is uncertain whether reductions in polygyny were motivated by HIV/AIDS, other prevailing sociocultural changes, or both. Importantly, we did not find that polygyny was being replaced by other more informal long-term concurrency (which has come to be referred to colloquially as “small houses” in Zimbabwe), as some have feared when speculating about the consequences of declines in polygyny for HIV transmission. As noted, the trend in polygyny was very similar to nonmarital concurrency and multiple partnerships over the first 3 survey rounds, but polygyny declined more steeply between rounds 3 and 4 and rebounded slightly in round 5. Round 4, which collected data from 2006 to 2008, occurred during the peak of the economic crisis in Zimbabwe, which could have affected men's ability to support multiple wives.

Polygyny is a complex concept, particularly in settings in southern and eastern Africa, where contemporary concepts of marriage are an intersection of traditional, religious, and civil institutions. Our definition of polygyny, based on a self-assessed question about the number of spouses, is intended to be inclusive of different definitions of marriage, without specifying the occurrence of religious ceremonies, civil registration, or traditional cultural practices, such as the payment of lobola (bridewealth). Evaluating whether these different institutions are particularly associated with higher levels of polygyny, divorce, or extramarital partnerships through additional qualitative and quantitative research may further nuance the policy implications of this study. Polygyny was much higher among men affiliated with traditional or apostolic religions or with no religious affiliation. The 12 study sites included in the Manicaland HIV/STD Prevention Project cohort do not include a representative population of the strict Apostolic religious sects, who condone polygyny but strictly bar casual relationships, alcohol consumption, and modern medicine, and thus might exhibit a different relationship between polygyny and HIV risk than the religions represented in this study [29].

Self-reported sexual behavior is susceptible to reporting bias [30]. To minimize this, we used the informal confidential voting interview method for collecting sex partnership information [19]. Since an increasing fraction of interviews used ICVI in each survey round, our results may underestimate the true change in risk behaviors over time. Nonetheless, this remains a limitation of our study, particularly if reporting biases have changed over time. Misreporting of retrospective dates of marriage or divorce could affect longitudinal analyses. For example, a crude value of 2.5% of unmarried men reported having experienced a divorce or separation at the next survey round (Table 1). These could occur when both a marriage and divorce occurred in the intersurvey period, or they could also be examples of misreporting dates of divorce as during the intersurvey period when they actually occurred before the previous survey.

To define the contribution of changes in the different types of partnerships to reducing incidence in Zimbabwe requires further epidemiological analysis and modeling and will depend on other characteristics in addition to the prevalence of the partnership types alone, such as the frequency of unprotected intercourse within the partnerships [14, 31] and the likelihood that persons entering different forms of partnership will be HIV positive. However, from this analysis, it is clear that the behavior change leading to the declines was not dominated by reductions in any single type of partnership, and there was no evidence of compensatory increases in some partnership types as others decreased. Because of the increased risk of dissolution and extramarital partnerships within polygynous unions, we do not recommend that polygyny be considered a safe form of concurrency separately from nonmarital concurrency in eastern Zimbabwe; rather, we recommend that polygynous men should be included as a population for further HIV prevention.

## Supplementary Data

Supplementary materials are available at *The Journal of Infectious Diseases* online (http://jid.oxfordjournals.org). Supplementary materials consist of data provided by the author that are published to benefit the reader. The posted materials are not copyedited. The contents of all supplementary data are the sole responsibility of the authors. Questions or messages regarding errors should be addressed to the author.

Supplementary Data
