# Supplementary material for: Trends in Concurrency, Polygyny, and Multiple Sex Partnerships During a Decade of Declining HIV Prevalence in Eastern Zimbabwe
Source: J Infect Dis. 2014 Dec 1;210(Suppl 2):S562–8. doi: 10.1093/infdis/jiu415 (PMC4231639; doi:10.1093/infdis/jiu415)
Supplement: Supplementary Data [file supp_210_suppl-2_S562__index.html]

Trends in Concurrency, Polygyny, and Multiple Sex Partnerships During a Decade of Declining HIV Prevalence in Eastern Zimbabwe — Supplementary Data 

# Trends in Concurrency, Polygyny, and Multiple Sex Partnerships During a Decade of Declining HIV Prevalence in Eastern Zimbabwe

## Supplementary Data

Supplementary Data

**Files in this Data Supplement:**

- Supplementary Data - Docx file
