# Supplementary material for: Trends in Concurrency, Polygyny, and Multiple Sex Partnerships During a Decade of Declining HIV Prevalence in Eastern Zimbabwe
Source: J Infect Dis. 2014 Dec 1;210(Suppl 2):S562–8. doi: 10.1093/infdis/jiu415 (PMC4231639; doi:10.1093/infdis/jiu415)
Supplement: Supplementary Data [file supp_jiu415_jiu415supp.docx]

**Supplementary Information**

Figure S1: The percentage of all male concurrency that is fully accounted for by polygyny in each survey round.


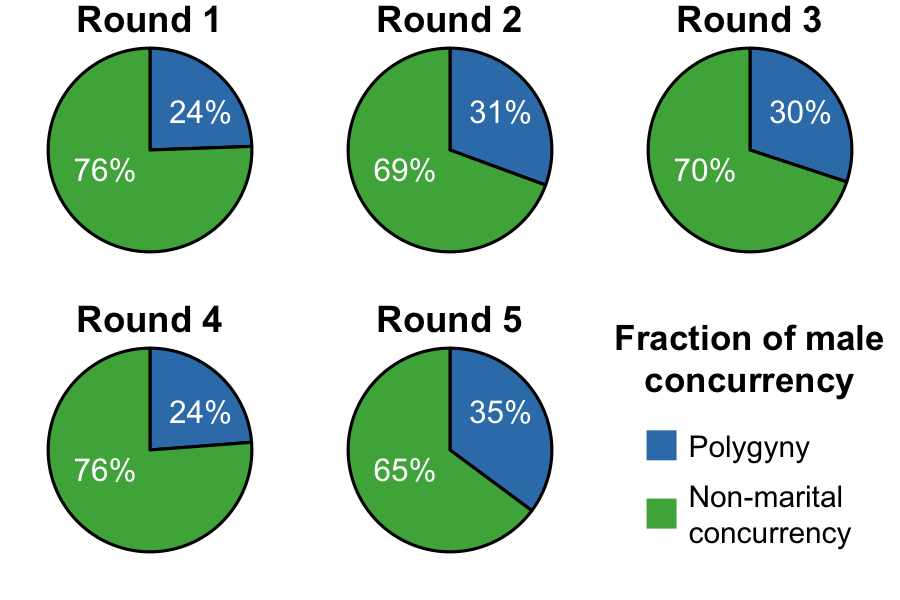


Table S1: Demographic characteristics of respondents by survey round

|  | **Round 1 (1998–2000)** | | **Round 2 (2001–2003)** | | **Round 3 (2003–2005)** | | **Round 4 (2006–2008)** | | **Round 5 (2009–2011)** | |
| --- | --- | --- | --- | --- | --- | --- | --- | --- | --- | --- |
|  | **Men** | **Women** | **Men** | **Women** | **Men** | **Women** | **Men** | **Women** | **Men** | **Women** |
| Total | 4267 | 5302 | 3140 | 4531 | 5733 | 9250 | 4386 | 6826 | 4656 | 7621 |
| Followed up at next round | 2066 | 3182 | 1735 | 2919 | 1665 | 3269 | 1847 | 3508 | – | – |
|  |  |  |  |  |  |  |  |  |  |  |
| Mean age  [IQR] | 27.6 [20–33] | 28.0 [20–36] | 28.0 [20–34] | 28.5 [19–38] | 28.8 [20–35] | 28.5 [20–36] | 29.1 [21–35] | 28.7 [20–36] | 30.4 [21–38] | 29.2 [21–37] |
|  |  |  |  |  |  |  |  |  |  |  |
| **Socioeconomic stratum** |  |  |  |  |  |  |  |  |  |  |
| Subsistence farming | 28.0% | 38.2% | 29.3% | 38.9% | 30.0% | 37.4% | 33.0% | 39.0% | 32.8% | 37.6% |
| Roadside trading | 12.8% | 20.3% | 14.3% | 20.6% | 17.0% | 20.1% | 18.8% | 19.9% | 19.7% | 20.0% |
| Agricultural estate | 40.3% | 26.7% | 39.8% | 27.3% | 35.2% | 27.6% | 29.9% | 26.3% | 29.2% | 24.8% |
| Commercial center | 19.0% | 14.7% | 16.5% | 13.1% | 17.7% | 14.9% | 18.3% | 14.8% | 18.3% | 17.6% |
|  |  |  |  |  |  |  |  |  |  |  |
| **Religion** |  |  |  |  |  |  |  |  |  |  |
| Christian | 53.4% | 70.0% | 60.2% | 71.7% | 58.9% | 67.1% | 49.5% | 52.0% | 47.0% | 49.5% |
| Traditional | 18.0% | 3.3% | 4.7% | 1.3% | 2.8% | 1.5% | 3.6% | 1.2% | 1.7% | 0.4% |
| Spiritual | 13.5% | 15.6% | 15.9% | 20.0% | 17.7% | 24.3% | 19.2% | 25.9% | 24.8% | 34.5% |
| Other | 5.0% | 6.0% | 3.2% | 3.0% | 2.5% | 2.6% | 12.5% | 17.4% | 11.5% | 13.6% |
| None | 10.1% | 5.0% | 16.0% | 4.0% | 18.1% | 4.5% | 15.3% | 3.5% | 15.0% | 1.9% |
|  |  |  |  |  |  |  |  |  |  |  |
| Married | 42.5% | 56.3% | 48.2% | 54.5% | 51.3% | 56.2% | 52.6% | 57.6% | 58.4% | 62.6% |

Table S2: The relative reduction in multiple partnerships, concurrency, and polygyny over survey rounds, adjusting for age-group, socioeconomic stratum, and religious affiliation

|  | **Men, 17–54 years** | | | **Women, 15–49 years** | |
| --- | --- | --- | --- | --- | --- |
|  | Multiple partnerships | Non-marital concurrency | Polygyny | Multiple partnerships | Concurrency |
|  |  |  |  |  |  |
| **Age-group** |  |  |  |  |  |
| 17–19 (M) / 15–19 (F) | 0.42 (0.38-0.47) | 0.65 (0.54-0.77) | 0.03 (0.01-0.08) | 0.32 (0.24-0.42) | 0.46 (0.29-0.74) |
| 20–24 | 0.99 (0.93-1.06) | 1.20 (1.04-1.39) | 0.25 (0.18-0.36) | 0.93 (0.76-1.13) | 0.98 (0.66-1.46) |
| 25–29 [Reference] | 1 | 1 | 1 | 1 | 1 |
| 30–34 | 0.86 (0.79-0.94) | 0.85 (0.70-1.02) | 1.25 (0.97-1.61) | 1.17 (0.95-1.44) | 1.80 (1.24-2.62) |
| 35–39 | 0.78 (0.71-0.86) | 0.85 (0.69-1.05) | 1.57 (1.21-2.03) | 0.88 (0.70-1.11) | 0.98 (0.62-1.54) |
| 40–44 | 0.65 (0.58-0.74) | 0.47 (0.35-0.63) | 1.71 (1.30-2.25) | 0.50 (0.37-0.67) | 0.60 (0.35-1.05) |
| 45–49 | 0.68 (0.60-0.78) | 0.58 (0.43-0.78) | 2.70 (2.09-3.50) | 0.37 (0.25-0.56) | 0.56 (0.27-1.14) |
| 50–54 | 0.59 (0.50-0.69) | 0.40 (0.27-0.58) | 2.46 (1.87-3.25) | — | ­— |
|  |  |  |  |  |  |
| **Socioeconomic stratum** |  |  |  |  |  |
| Subsistence farming [Ref] | 1 | 1 | 1 | 1 | 1 |
| Roadside trading | 0.93 (0.85-1.01) | 0.97 (0.81-1.15) | 0.53 (0.35-0.80) | 0.98 (0.75-1.27) | 1.21 (0.78-1.88) |
| Agricultural estate | 1.11 (1.04-1.19) | 1.18 (1.03-1.35) | 1.87 (1.51-2.32) | 1.90 (1.56-2.32) | 1.51 (1.03-2.22) |
| Commercial center | 1.22 (1.13-1.32) | 1.23 (1.05-1.43) | 1.39 (1.08-1.78) | 3.67 (3.03-4.45) | 4.35 (3.08-6.14) |
|  |  |  |  |  |  |
| **Religious affiliation** |  |  |  |  |  |
| Christian [Reference] | 1 | 1 | 1 | 1 | 1 |
| Traditional | 1.16 (1.06-1.27) | 0.87 (0.71-1.07) | 1.80 (1.40-2.30) | 1.50 (1.02-2.21) | 1.40 (0.69-2.85) |
| Spiritual | 0.86 (0.80-0.93) | 0.90 (0.77-1.04) | 1.54 (1.24-1.92) | 0.88 (0.73-1.06) | 0.54 (0.36-0.80) |
| Other | 0.88 (0.78-1.00) | 0.85 (0.67-1.08) | 1.39 (0.97-1.99) | 0.78 (0.58-1.06) | 0.83 (0.50-1.39) |
| None | 1.31 (1.23-1.40) | 1.23 (1.07-1.42) | 2.00 (1.63-2.45) | 3.15 (2.61-3.80) | 3.59 (2.57-5.01) |
|  |  |  |  |  |  |
| **Survey round** |  |  |  |  |  |
| Round 1 [Reference] | 1 | 1 | 1 | 1 | 1 |
| Round 2 | 0.63 (0.58-0.68) | 0.50 (0.42-0.59) | 0.66 (0.52-0.83) | 0.69 (0.56-0.85) | 0.58 (0.40-0.83) |
| Round 3 | 0.62 (0.58-0.67) | 0.45 (0.39-0.52) | 0.57 (0.46-0.71) | 0.47 (0.39-0.56) | 0.27 (0.19-0.38) |
| Round 4 | 0.43 (0.40-0.47) | 0.37 (0.32-0.44) | 0.31 (0.23-0.41) | 0.43 (0.35-0.53) | 0.30 (0.20-0.44) |
| Round 5 | 0.34 (0.31-0.38) | 0.30 (0.25-0.36) | 0.42 (0.33-0.53) | 0.32 (0.26-0.41) | 0.29 (0.20-0.44) |

Estimates and 95% CIs based on log-binomial models adjusting for all other covariates listed in the table.
